# Bis-(di-4-phenyl-benzylaminethiocarbonyl)disulfide sensitizes ABCC2/ALDH3A1 overexpressing NSCLC cells to cisplatin

**DOI:** 10.1080/15384047.2026.2683169

**Published:** 2026-07-20

**Authors:** Jolanta Kryczka, Jakub Mateusz Kryczka, Łukasz Janczewski, Sho Shimida, Andrzej Frączyk, Beata Kolesińska, Joanna Boncela, Ewa Brzeziańska-Lasota

**Affiliations:** a Department of Biomedicine and Genetics, Medical University of Lodz, Lodz, Poland; b Laboratory of Cell Signalling, Institute of Medical Biology, Polish Academy of Sciences, Lodz, Poland; c Institute of Organic Chemistry, Faculty of Chemistry, Lodz University of Technology, Lodz, Poland; d Faculty of Science and Technology, Keio University – Keio Gijuku Daigaku, Kohoku, Yokohama, Japan; e Institute of Applied Computer Science, Lodz University of Technology, Lodz, Poland

**Keywords:** Non-small cell lung cancer, NSCLC, cisplatin, chemo-resistance, cisplatin-resistance, ALDH, ABC

## Abstract

**Background:**

Lung cancer presents complex etiopathology involving a mix of genetic predispositions and environmental factors. The best treatment modality is surgical resection. However, it becomes ineffective in the advanced metastatic stage. Thus, cisplatin-based chemotherapy, though restricted by an intrinsic and/or acquired chemo-resistant phenotype, remains the first-line therapy for advanced non-small-cell cancer (NSCLC).

**Methods:**

Various tools were used to verify the mRNA expression and protein levels of ABC and ALDH proteins in patient-derived non-small-cell lung cancer (NSCLC) samples (GSE102287, GSE43580) and cell lines A549 and NCI-H158 (including their respective cisplatin-resistant variants) to verify the cisplatin-sensitizing abilities of newly synthesized bis-(di-4-phenyl-benzylaminethiocarbonyl)disulfide—non-toxic ALDH and ABCC2 inhibitors.

**Results:**

We identified significant molecular differences in the expression levels of ABCC1, ABCC5, ABCC3, ALDH7A1, and ALDH3A1. Additionally, a fairly significant patient subgroup (Z-score > 1.5), characterized by ABCC2 and ALDH3A1 overexpression, was identified. Importantly, *in vitro,* ABCC2 and ALDH3A1 accompany the acquisition of cisplatin resistance in A549 cells. Bis-(di-4-phenyl-benzylaminethiocarbonyl)disulfide reverses cisplatin resistance in a cisplatin-resistant variant of A549 cells (via ALDH and ABCC2 inhibition) but not in NCI-H158 cells.

**Conclusions:**

Molecular categorization of NSCLC cancer is essential for predicting therapy outcomes, enabling the use of bis-(di-4-phenyl-benzylaminethiocarbonyl)disulfide as a cisplatin therapy enhancer for NSCLC patients' subpopulation with significant ABCC2 and ALDH3A1 overexpression.

## Graphical abstract

**Figure:**
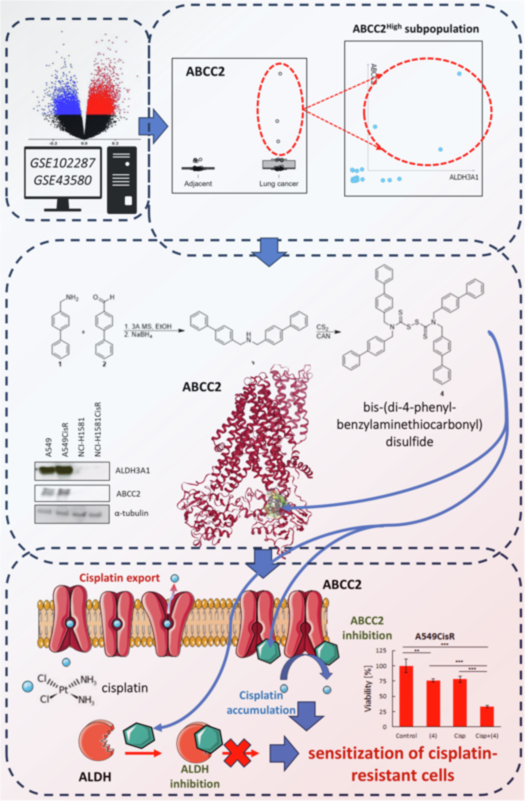

## Introduction

Lung cancer is one of the most diagnosed types of cancer, accounting for nearly two million deaths worldwide each year.^1^ Its complex etiopathology involves a mix of genetic predispositions and environmental factors.^2^ ^,^ ^3^ Based on histopathological determination, lung cancer is categorized into two main histological groups: small cell lung cancer (SCLC) and non-small cell lung cancer (NSCLC), which accounts for approximately 80–85% of all lung cancer cases. NSCLC comprises several histological subtypes: adenocarcinoma (AC, 40–50% cases), squamous cell carcinoma (SCC, 20–30% cases), and large cell lung carcinoma (LCLC, 10%–15% cases).^2^ ^,^ ^4^ ^,^ ^5^ AC is more frequent in women than SCC (55% vs 25%), and in men's case, this trend is reversed (30% vs 57%), whereas LCLC frequency is comparable.^6^ The best treatment modality (according to the American Society of Clinical Oncology) to improve the long-term survival of stage I and II (according to the American Joint Committee on Cancer (AJCC) classification) lung cancer patients is surgical resection of the tumor and mediastinal lymph nodes.^7^ Unfortunately, a large percentage of NSCLC patients (47%) are diagnosed at advanced stages (III/IV), when the tumor has already spread to lymph nodes or distant organs, rendering surgical resection ineffective.^8^ Thus, surgical resection and chemotherapy remain one of the major treatment methods, with cisplatin being the most popular chemotherapy agent.^3^ However, exposure to cisplatin may trigger a multipronged adaptive response of NSCLC cells, leading to the acquisition of a highly therapy-resistant (chemo-resistant) phenotype.^1^ Importantly, resistance to cisplatin is a patient-specific mix of many mechanisms, such as enhanced drug metabolism and export (mainly mediated by proteins belonging to the ATP-binding cassette—ABC proteins family), diminished drug influx, promotion of DNA damage response, increased anti-apoptotic signaling, etc.^1,9‐11^. Additionally, intrinsic chemoresistance mechanisms already present in chemotherapy-naïve cancer cells have been reported. They are often related to the cancer stem cell (CSC) phenotype, epithelial-to-mesenchymal transition (EMT), and hypoxia-related metabolic switch toward aerobic glycolysis, known as the “Warburg effect”.^12‐15^ Recently, to describe the non-binary process of the acquisition of drug resistance, which develops through trajectories of cell-state transitions accompanied by a progressive increase in cell fitness, the “resistance continuum” term was proposed by França et al.^16^. This concept highlights the fact that gradual exposure to toxic conditions can improve (or tune) the overall survival of a cell population by allowing cells to accumulate transcriptional and epigenetic changes, leading to stable resistance through several intermediate adaptive states. Additionally, the molecular heterogeneity of advanced NSCLC subtypes may significantly impact therapy outcomes, which could be analyzed and potentially predicted by a combination of precise, pre-selected markers.^3^ ^,^ ^17^ Our recent study proved that supplementation with synthetic isothiocyanates ITCs—(2-isothiocyanatoethane-1,1-diyl)dibenzene, 1-(isothiocyanatomethyl)-4-phenylbenzene or 1-isothiocyanato-3,5-bis(trifluoromethyl)benzene—significantly decreased cisplatin-resistance and the invasive potential of cisplatin-resistant NSCLC models (A549), reversing EMT toward an epithelial phenotype.^18^ These results proved that proper histological and molecular categorization, which may improve therapy planning and outcomes for NSCLC patients, is urgently needed. In this study, we reasoned that markers that are potentially useful for the molecular categorization of NSCLC and for the prediction of therapy outcome should be searched among proteins involved in acquired and intrinsic drug resistance. Thus, we have focused on ABC proteins—major drug exporters, markers of chemotherapy resistance acquisition, and ALDH isoforms such as ALDH1A1, ALDH3A1, and ALDH1A7, which are considered CSC markers.^18‐20^ Importantly, clinically relevant NSCLC subgroups are often defined by ABCC/ALDH expression patterns, and ABCC2/ALDH3A1^high^ NSCLC could be sensitized against cisplatin by an ALDH/ABCC2-targeting compound. Thus, to validate our hypothesis and test chosen markers, we synthesized a derivative of a previously tested potent ALDH inhibitor: 1-(isothiocyanatomethyl)-4-phenylbenzene,^18^ which combines two such structures via disulfide-bound, named bis-(di-4-phenyl-benzylaminethiocarbonyl)disulfide, and tested its anticancer potential toward cisplatin-resistant variants of NSCLC cell lines.

## Results

### ABC and ALDH mRNA expression pattern in NSCLC and adjacent, noncancerous lung tissue

**Figure 1.**
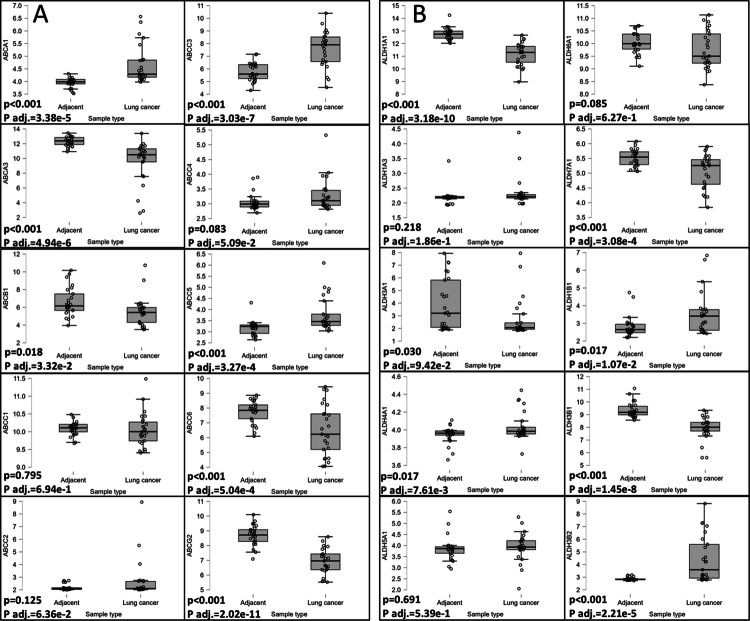
Differences in mRNA expression of genes coding for ABC (A) and ALDH (B) proteins between lung cancer samples (LC) and adjacent tissue (Adj.). Transcriptomic data were downloaded from the GEO database (GSE102287). A paired Student's *t*-test (*n* = 25 LC/Adj. pairs) calculation and visualization via a ladder graph performed using JASP 0.16.0.0, *p*-value, and the Benjamini and Hochberg adjusted *p*-value (*p* adj.) are presented in each subfigure.

For several decades, ABC proteins have been closely related to cancer progression and the acquisition of drug resistance. However, in contrast to *in vitro* studies, which are based on cancer cell lines, ABC protein function and molecular level in cancer cells *in vivo* are far more complicated and elusive.^21^ Thus, to analyze the mRNA expression level of major drug resistance-related ABC proteins ABCB1, ABCC1-6, ABCG2 (xenobiotic exporters), and known cisplatin transporters ABCA1 and ABCA3, data from the GEO database (GSE102287) were downloaded. This dataset consists of 66 samples of non-small lung cancer (adenoma) and adjacent noncancerous lung tissue. Fifty paired samples (25 lung cancer samples named LC and 25 corresponding adjacent noncancerous tissue samples named Adj.) The data were analyzed using paired Student's *t*-tests and visualized using Jasp 0.16.0.0 software; the results are presented in Figure 1. Lung cancer samples presented significantly upregulated expression levels of mRNA coding ABCA1, ABCC3, and ABCC5 in comparison to adjacent noncancerous lung tissue. Interestingly, the mRNA levels of ABCA3, ABCC6, and ABCG2 were significantly lower in the corresponding adjacent tissue. Recent studies proved that different chemotherapy schemes are often ineffective against cancer cells characterized by high protein levels of ALDH. Additionally, several ALDH isoforms, such as ALDH1A1, ALDH1A3, ALDH1A7, ALDH3A1, ALDH4A1, ALDH5A1, and ALDH6A1, are considered to be cancer stem cell (CSC) markers.^18‐20^ Thus, the mRNA expression levels of aldehyde dehydrogenase (ALDH) isoforms were analyzed in the same datasets and are presented in Figure 1. Our analysis shows that ALDH4A1, ALDH1B1, and ALDH3B2 are significantly upregulated in lung cancer samples compared to the adjacent noncancerous tissue. On the other hand, two of the CSC markers—ALDH1A1, and ALDH3A1, followed by one other isoform: ALDH3B1, presented significantly decreased expression levels in non-small lung cancer samples compared to (control) noncancerous tissue.

### ABC and ALDH mRNA expression pattern in NSCLC subtypes

**Figure 2.**
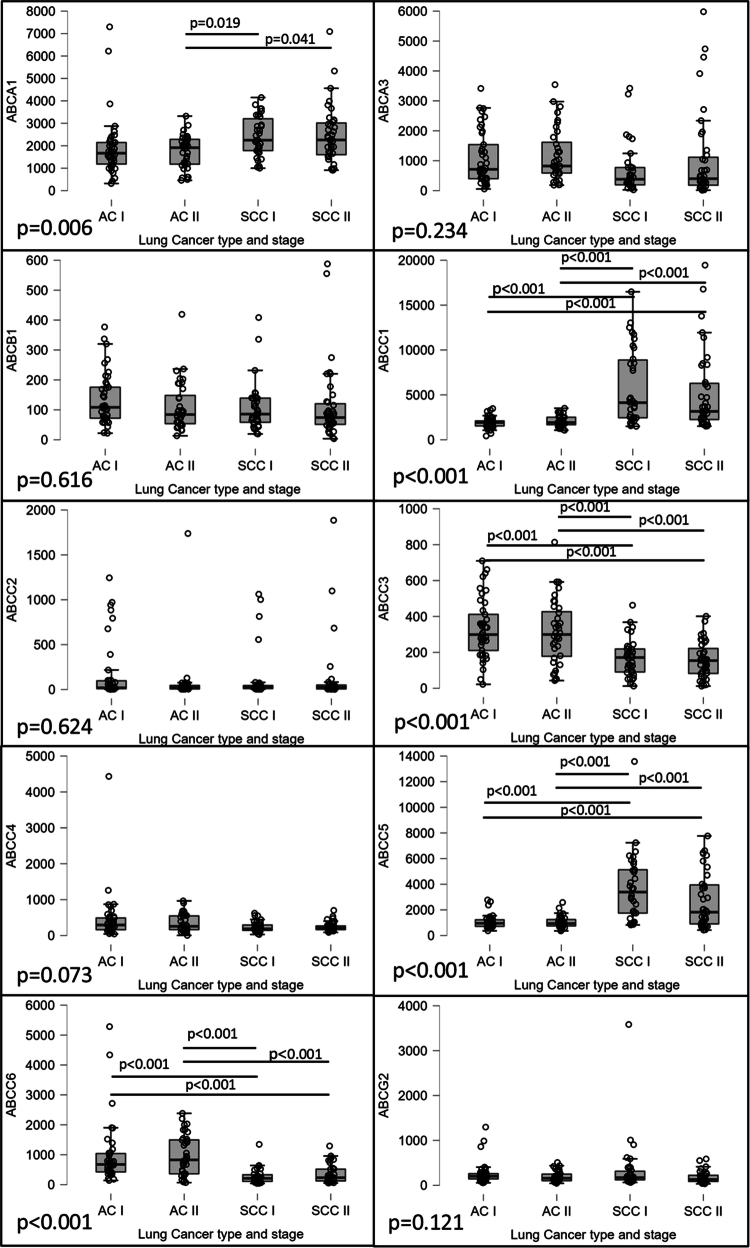
mRNA expression of genes coding ABC proteins in stages I and II of NSCLC adenocarcinoma (AC) and squamous carcinoma (SCC) subtypes. Transcriptomic data were downloaded from the GEO database (GSE43580). Statistical differences between AC (I and II) and SCC (I and II) lung cancer groups were analyzed via a normality test (Shapiro–Wilk) followed by one-way ANOVA post hoc test (Tukey) and visualized using Jasp 0.16.0.0 software; *p*-values are presented in each subfigure.

To verify the mRNA expression patterns of the analyzed genes regarding the different NSCLC types and stages, data from the GSE43580 dataset were downloaded. This dataset consists of 150 NSCLC samples—77 adenocarcinoma (AC) including *n* = 41 Stage I and *n* = 36 Stage II and 73 squamous cell carcinoma (SCC) *n* = 34 Stage I and *n* = 39 Stage II. Differences in mRNA expression level patterns between AC (I and II) and SCC (I and II) groups were analyzed via one-way ANOVA post hoc test (Tukey) and visualized using Jasp 0.16.0.0 software (Figures 2 and 4). Neither ABC nor ALDH mRNA expression was related to cancer stage in both AC and SCC groups. However, each AC and SCC lung cancer type presented specific mRNA expression patterns for ABCA1, ABCC1, ABCC3, ABCC5, and ABCC6 (Figure 2).

**Figure 3.**
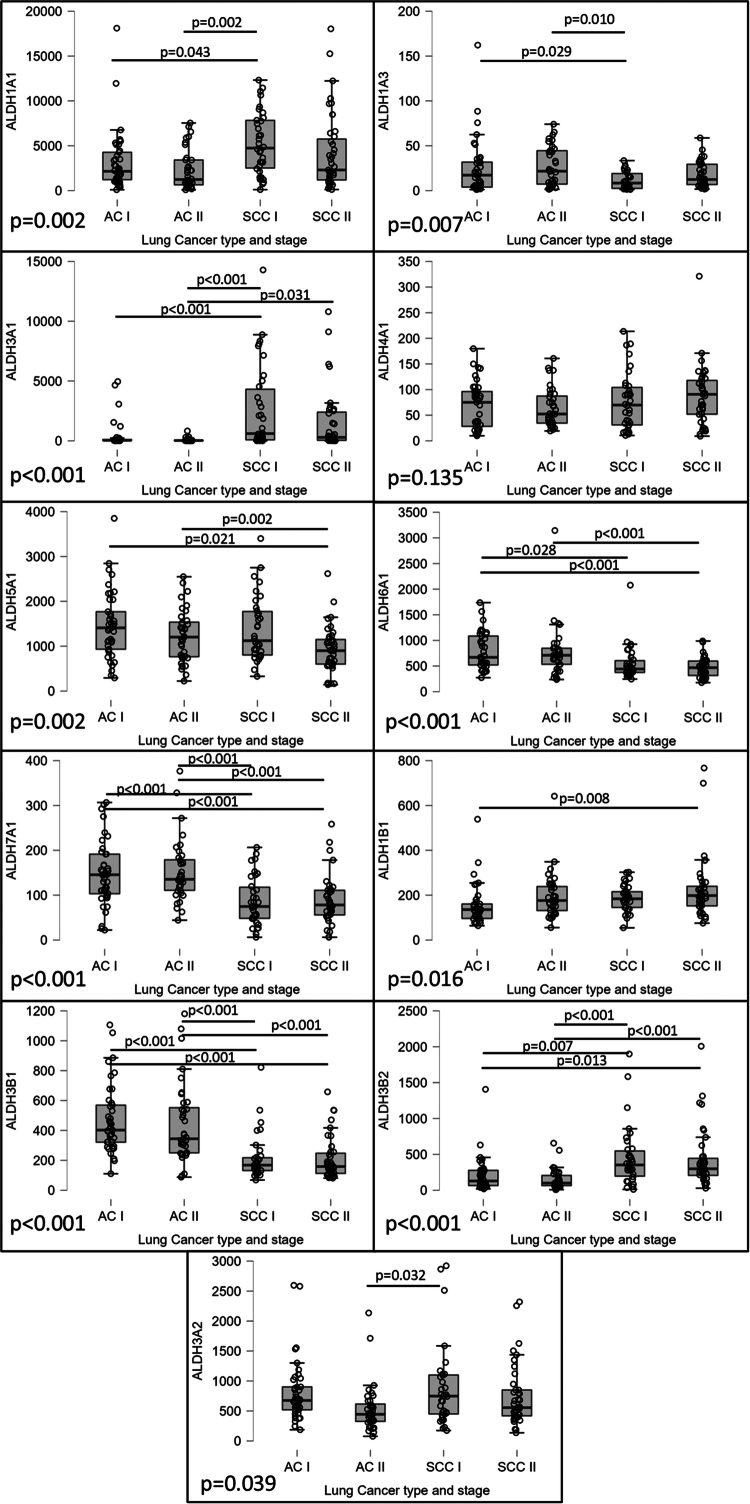
mRNA expression of genes coding ALDH proteins in stages I and II of NSCLC adenocarcinoma (AC) and squamous carcinoma (SCC) subtypes. Transcriptomic data were downloaded from the GEO database GSE43580. Statistical differences between AC (I and II) and SCC (I and II) lung cancer groups were analyzed via a normality test (Shapiro–Wilk) followed by one-way ANOVA post hoc test (Tukey) and visualized using Jasp 0.16.0.0 software; *p*-values are presented in each subfigure.

AC group presented higher expression levels of ABCC3 and ABCC6 and lower of ABCC1 and ABCC5 compared to SCC group. Additionally, ABCA1 expression is significantly higher in SCC compared to AC in stage II of the tumor (AJCC), but such a difference was not observed in stage I. A created panel of ALDH isoform mRNA expression proved that none can be used to distinguish between stage I and stage II in either AC or SCC group; however, substantial differences between the analyzed lung cancer histological subtypes were noticed. ALDH6A1, ALDH7A1, and ALDH3B1 presented higher mRNA expression levels, whereas ALDH3A1 and ALDH3B2 have lower mRNA expression in the AC group compared to the SCC group (Figure 3). Interestingly, ALDH1A1 mRNA expression is lower in Stage I and Stage II of the AC group compared to the SCC group in Stage I but not in Stage II.

### Identification and characteristics of ABCC2^High^subgroups

**Figure 4.**
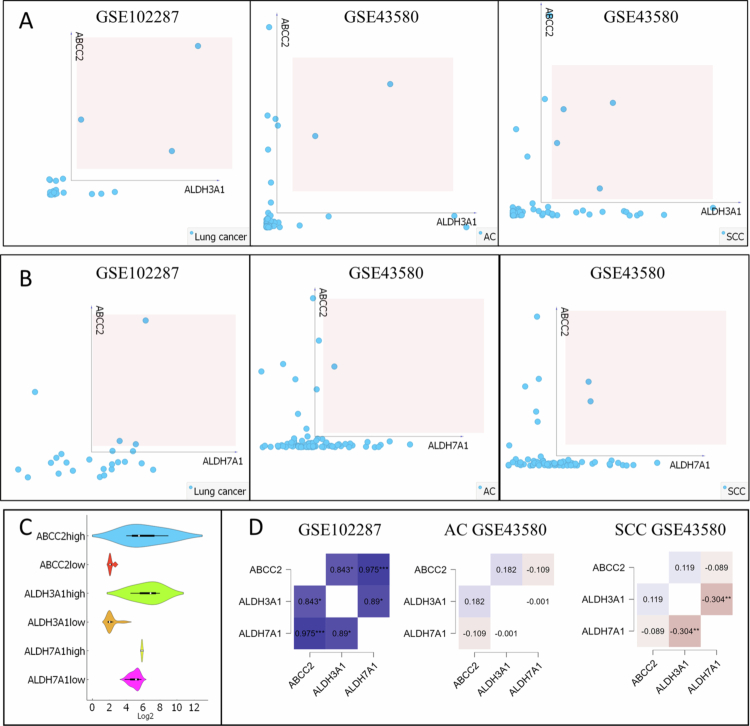
ABCC2^High^ subpopulation characteristics in NSCLC patient samples. mRNA level mutual relationships of ABCC2 and ALDH3A1 (A) and ALDH7A1 (B) in NSCLC patients (lung cancer) and major NSCLC histotype adenocarcinoma (AC) and squamous carcinoma (SCC). 2D visualization was performed using linear projection and the RadViz algorithm of the Orange Data Mining software. The pink square represents the data with the best match in the I quadrant of the Cartesian coordinate system, where the projection values for X- and Y-axes are >0. Violin plot of log2-transformed mRNA expression levels of ABCC2, ALDH3A1, and ALDH7A1 in respective high and low subpopulations (Z-score > 1.5) of NSCLC, visualization was performed via Orange Data Mining software (kernel: normal, scale: width) (C). Pearson correlation matrix of ABCC2, ALDH3A1 and ALDH7A1 genes mRNA levels (D) using JASP 0.16.0.0 software; *p < 0.05, **p < 0.005, and ***p < 0.001. Transcriptomic data downloaded from the GEO database, GSE102287 and GSE43580 datasets.

Further analysis of mRNA expression patterns of ABC proteins in patient samples proved that, despite no significant changes in the expression level of major cisplatin exporters—ABCC2—between LC and noncancerous adjacent tissue (GSE102287), as well as between AC and SCC histotypes (GSE43580), several outlying data points, creating significant subgroups characterized by high ABCC2 level, could be distinguished with GSE102287 ABCC2 Z-score > 1.5 (3.76; 1.63), and GSE43580 ABCC2 Z-score > 2 (AC: 5.12; 3.55; 2.68; 2.59; 2.41; 2.12 and SCC: 2.75; 2.24; 2.22; 2.19; 2.02). Importantly, recent data suggest that targeting ABCC2, thereby down-regulating its expression at the cytomembrane, may enhance platinum-based chemotherapy and reduce adverse drug reactions (ADRs).^22^ ^,^ ^23^ We verified that ABCC2^High^ subpopulations using the linear projections component of the RadViz algorithm via the Orange Data mining platform and noticed that in several patient samples, ALDH3A1 (Figure 4A) and ALDH7A1 (Figure 4B) expression is also significantly upregulated, especially in the case of SCC NSCLC. This method is based on the VizRank algorithm, introduced by Gregor Leban in 2006, which is a systematic method for evaluating low-dimensional projections of high-dimensional data with respect to their ability to visually separate classes.^24^ Unlike classical dimensionality reduction methods (e.g., principal component analysis), the linear projection component of the RadViz algorithm does not optimize a global objective. Instead, it performs a heuristic search over a discrete set of candidate projections. Representation of data with the best-matching data (the highest projection fit) are visualized in the I quadrant of the Cartesian coordinate system (pink square in Figure 4A,B), and the worst-matching data with the lowest projection fit in the III quadrant. Data distributions for the respective high and low subpopulations of ABCC2, ALDH3A1, and ALDH7A1 in NSCLC (Z-score > 1.5), visualized as violin plots of log2-transformed pooled data, further confirmed our hypothesis (Figure 4C). Interestingly, the mRNA expression of ALDH3A1 and ALDH7A1 was significantly lower in the lung cancer samples compared to adjacent tissue, and considering pooled LC and adjacent tissue samples (GSE102287), positively correlated with ABCC2 mRNA expression level (Figure 4D). On the other hand, the mutual relationships of the mRNA expression levels of ABCC2 and ALDH3A1 present no significant correlation in AC histotype and a negative correlation in SCC histotype (Figure 4D). This observation highlights the necessity of individual molecular analysis for proper therapy planning.

### ABC and ALDH protein levels in NSCLC cancer samples

**Figure 5.**
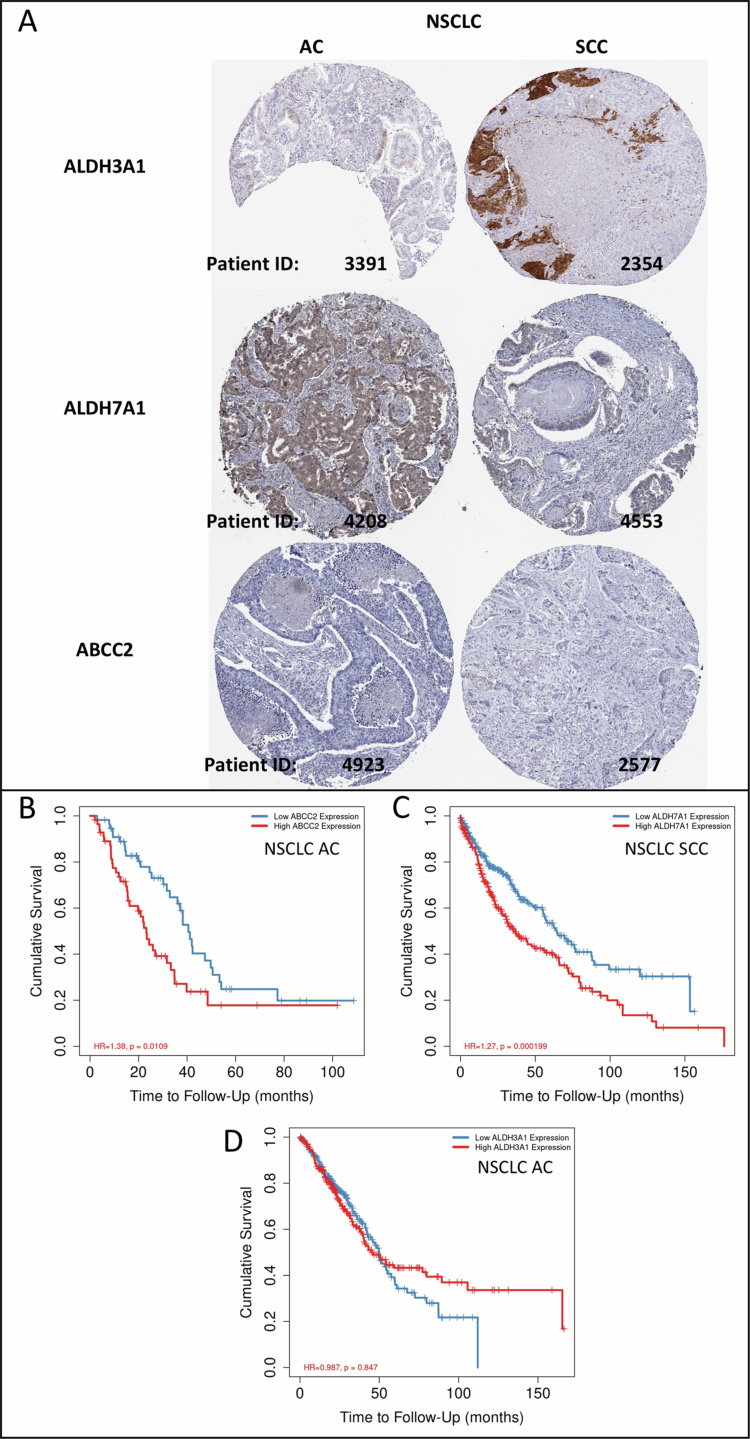
Representative image of ALDH3A1, ALDH7A1, and ABCC2 protein detection in NSCLC tissue samples (A). Human lung adenocarcinoma (AC) and squamous carcinoma (SCC) tissue samples were stained with antibodies: anti-ALDH3A1-HPA051150, anti-ALDH7A1-HPA053675, and anti-ABCC2-HPA071145. The patients' ID numbers are presented in each subfigure. Image credits: Human Protein Atlas, www.proteinatlas.org. ABCC2 (B), ALDH7A1 (C), and ALDH3A1 (D) mRNA expression impact on NSCLC patients' survival rate. ABCC2, ALDH7A, and ADLH3A1 impact on the survival rate of NSCLC AC (*n* = 497) and NSCLC SCC (*n* = 489) (respectively) was analyzed using TCGA data and visualized by the TIMER2.0 platform with log-rank test score *p* = 3.63 × 10^−6^ (ABCC2) and *p* = 6.74 × 10^−4^ (ALDH7A1).

To validate our mRNA-based analysis, we verified the protein levels of ALDH3A1, ALDH7A1, and ABCC2 in adenocarcinoma and squamous cell carcinoma variants of NSCLC. The Human Protein Atlas database was employed. The analyzed lung cancer samples were stained with antibodies (anti-ALDH3A1-HPA051150, anti-ALDH7A1-HPA053675, and anti-ABCC2-HPA071145).^25‐27^ Tumor cell **s**taining intensity and cellular location are described in Table 1. Representative visualizations of cancer cell staining are presented in Figure 5A. Analogous to the mRNA-based observations, ALDH3A1 protein levels were significantly higher in the SCC subtype, whereas ALDH7A1 in the AC subtype. As ABCC2 mRNA expression was upregulated in a small patient group and its mean expression level in AC and SCC NSCLC was low, the ABCC2 protein was not detected in any of the NSCLC variants. Next, the impact of the mRNA level on NSCLC patient survival was analyzed using TCGA data and the TIMER2.0 platform.^28^ High expression levels of ABCC2 and ALDH7A1 are unfavorable factors for the respective histotypes of AC NSCLC (log-rank test score *p* = 3.63 × 10^−6^) (Figure 5B) and SCC NSCLC (log-rank test score *p* = 6.74 × 10^−4^) (Figure 5C). Interestingly, the mRNA expression level of ALDH3A1 is not a relevant factor in the 5-year (60 months) survival rate of AC NSCLC patients (log-rank test *p* = 4.27 × 10^−1^) (Figure 5D), presenting some moderately positive impact on long-term survival.

**Table 1.** Analysis of protein staining in NSCLC cancer samples. NSCLC—non-small cell lung cancer, AC—adenocarcinoma, SCC—squamous cell carcinoma, data collected from the Human Protein Atlas, pathology section.^26^ ^,^ ^27^

Protein	NSCLC type	Tumor cell staining	Location
ALDH3A1	AC	Weak	Cytoplasmic/membranous
	SCC	Strong	Cytoplasmic/membranous/nuclear
ALDH7A1	AC	Moderate	Cytoplasmic/membranous
	SCC	Not detected	Not detected
ABCC2	AC	Not detected	Not detected
	SCC	Not detected	Not detected

### ABC and ALDH protein levels in cisplatin-resistant and sensitive variants of lung cancer cell lines

**Figure 6.**
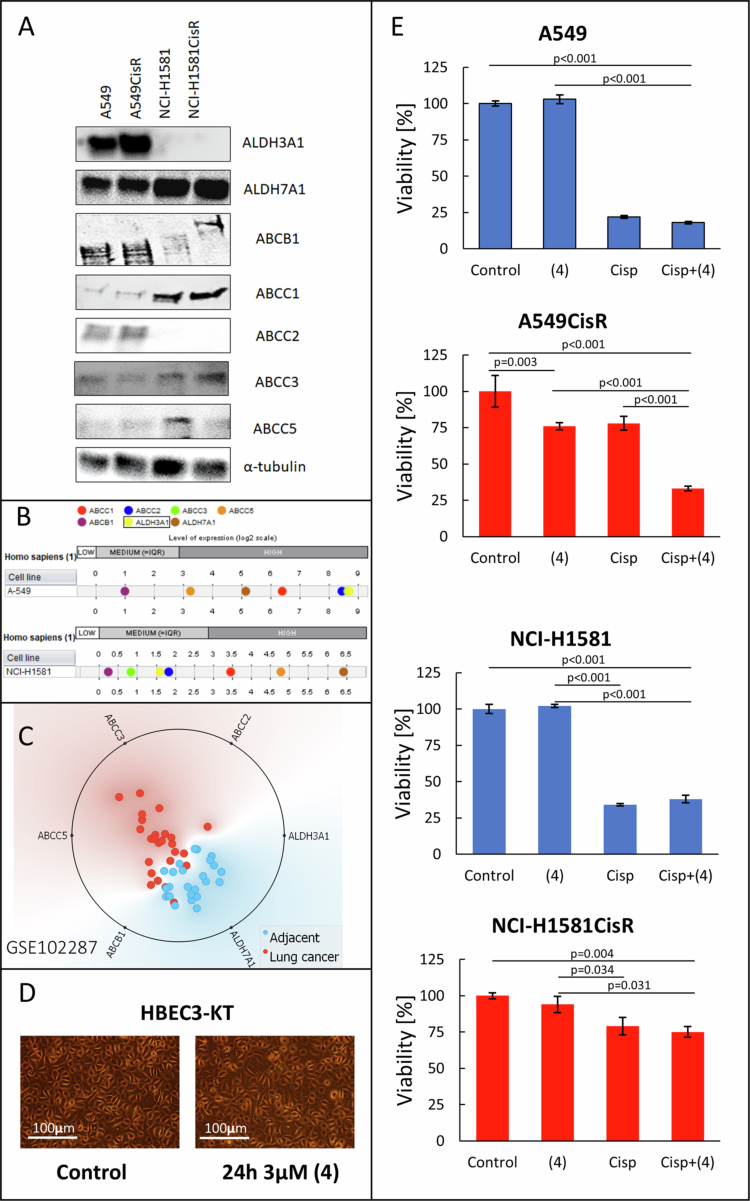
ABC and ALDH protein levels in Cisplatin-resistant and sensitive NSCLC cell lines. Western Blot (*n* = 3) analysis of ALDH3A1, ALDH7A1, ABCB1, ABCC1, ABCC2, ABCC3, and ABCC5 levels in NSCLC adenocarcinoma cell line A549 and its cisplatin-resistant variant A549CisR and in NSCLC large cell cancer cell line NCI-H1581 and its cisplatin-resistant variant NCI-H1581CisR (A). A549 and NCI-H1581 mRNA levels of *ALDH3A1, ALDH7A1, ABCB1, ABCC1, ABCC2, ABCC3,* and *ABCC5* were analyzed using GENEVESTIGATOR platform (B). Discriminative capabilities of chosen ABC and ALDH mRNA expression as attributes for RadViz algorithm (C), calculation and visualization based on transcriptomic data of lung cancer and adjacent tissue samples from GSE102287. Microscopic visualization of control HBEC3-KT cells treated with 3 µM bis-(di-4-phenyl-benzylaminethiocarbonyl)disulfide (**4**) after 24 h of incubation (D). Impact of cisplatin (**Cisp**) and 3 µM bis-(di-4-phenyl-benzylaminethiocarbonyl)disulfide (**4**) on A549, A549CisR, NCI-H1581 and NCI-H1581CisR viability measured by WST-1. The data were analyzed via a normality test (Shapiro–Wilk) followed by a one-way ANOVA post hoc test (Tukey) and visualized using Jasp 0.16.0.0 software, *p*-values are presented in each subfigure, *n* = 6 (E).

To verify potential differences in the protein levels of the previously analyzed ALDH isoforms (ALDH3A1 and ALDH7A1) and ABC (ABCB1, ABCC1, ABCC2, ABCC3, and ABCC5) in the NSCLC cell lines and their changes upon acquisition of cisplatin resistance, two cell lines were chosen: A549 (adenocarcinoma) and NCI-H1581 (stage 4 of large cell lung cancer). Cisplatin-resistant variants were obtained by constant culturing in increasing cisplatin (#15663-27-1, Sigma-Aldrich, St. Louis, MO, USA) concentrations (1–10 µM), as previously described in ref. 18 with A549 IC50 = 75 µM, A549CisR IC50 = 150 µM, NCI-H1581 IC50 = 28 µM, and NCI-H1581CisR IC50 = 50 µM.^18^ Our analysis showed that the protein levels of ALDH3A1 and ALDH7A1 were increased (approximately 25% and 11%, respectively) in A549CisR compared to the parental non-resistant variant (Figure 6A, Supplementary data S1). However, ALDH3A1 was barely detected in both NCI-H1581 variants, and ALDH7A1 was present at approximately 10% higher level in resistant NCI-H1581CisR. Importantly, A549CisR presents an approximately 25% higher level of the major cisplatin transporter ABCC2 than its parental cell line (Figure 6A, Supplementary data S1).^29^ One of the most prominent drug transporters—ABCB1—was significantly upregulated in NCI-H1581CisR compared to the parental NCI-H1581 as well as in both A549 variants compared to both variants of NCI-H1581, regarding a) the fully glycosylated form (~170 kDa) and b) the core-glycosylated or deglycosylated forms (~140–120 kDa).^30^ Additionally, in the A549 cell line, no significant changes in ABCC1 or ABCC5 expression were observed, with slight downregulation of ABCC3. Furthermore, the ABCC3 protein level in NCI-H1581CisR was approximately 40% higher than in the non-resistant variant NCI-H1581 and both variants of A549, whereas the ABCC5 level was found to be significantly greater (approximately 50%) in parental NCI-H1581 (Supplementary data S1). Next, the mRNA levels coding proteins present at different levels between the tested cell lines were verified using the GENEVESTIGATOR platform (Figure 6B). Analogous to our protein data, ALDH3A1 and ABCC2 present significantly higher, whereas ALDH7A1 presents lower mRNA levels in the A549 cell line compared to the NCI-H1581. Additionally, to further understand the impact of the chosen proteins on lung cancer, potential discriminating abilities were verified using the GSE102287 dataset, which consists of paired lung cancer samples and adjacent tissue via the RadViz algorithm and the Orange data mining software (Figure 6C). This visualization method is best described through a physical analogy involving a system of multiple springs, in which each spring connects an attribute (e.g., a selected gene) to a data point within the visualization space. The stiffness of each spring, in accordance with Hooke's law, is proportional to the corresponding attribute value (e.g., gene expression level).^24^ Consequently, attributes with higher values exert stronger attractive forces, drawing the data point closer. The final position of the data point is determined by the equilibrium condition in which the net force acting on the point equals zero (the vector sum of all spring forces is balanced); thus, the closer the position is situated to one or a set of attributes, the higher their value is compared to other attributes.^31^ The obtained data suggest that high mRNA expression of ALDH7A1 is related to non-cancerous adjacent tissue and thus probably a non-cisplatin-resistant phenotype, whereas ABCC3, ABCC5, ABCC2, and ALDH3A1 relate to NSCLC samples.

Following our previous work, regarding ITCs impact on the reversion of cisplatin resistance in NSCLC, and the promising effect of 1-(isothiocyanatomethyl)-4-phenylbenzene, we decided to combine two 1-(isothiocyanatomethyl)-4-phenylbenzene particles via S–S bond.^18^ Bis-(di-4-phenyl-benzylaminethiocarbonyl)disulfide (**4**) was obtained in a two-step reaction (method section). In the first step, described by Fang and Jacobsen, 4-phenylbenzylamine (**1**) and biphenyl-4-carboxaldehyde (**2**) were dissolved in abs. EtOH in the presence of 3 Å MS.^32^ The reaction was controlled by ^1^H NMR. After 2 h, the conversion of the aldehyde into the imine was >95%, and NaBH_4_ was added to reduce the imine to a secondary amine. Di-(4-phenylbenzyl)amine (**3**) was isolated by recrystallization (EtOAc/hexane) with 43% yield.

In the second step, based on Li and co-workers, amine **3** and CS_2_ were dissolved in THF in the presence of 0.05 mol% ceric ammonium nitrate (CAN) as a single-electron transfer (SET) catalyst.^33^ The reaction was stirred for 10 min with a stopper on the flask and then 8 h in an open flask using air as an oxidant. The final bis-(di-4-phenyl-benzylaminethiocarbonyl)disulfide (**4**) was isolated by column chromatography (hexane:EtOAc 3:1) with low yield but high purity (>99%). The structure of compound **4** (Experimental Section) was confirmed by ^1^H and ^13^C NMR spectra and LC-MS confirmed the mass.

Bis-(di-4-phenyl-benzylaminethiocarbonyl)disulfide (**4**) affinity to the ABC proteins and ALDH isoforms was calculated using AutoDock Vina, and the available crystal structures. The average affinity values of the analyzed and known (for reference) ligands for the selected proteins are presented in Table 2.

**Table 2.** The average values of the bis-(di-4-phenyl-benzylaminethiocarbonyl)disulfide (4) affinity for selected ALDH isoforms and ABC proteins. Protein structures were analyzed from the Protein Data Bank format (.PDB). For reference, the affinity values of known ligands to the analyzed structures are presented beneath each data section.

Protein	.PDB structure	Affinity [kcal/mol]
ALDH3A1	3sza	−6.20
	3szb	−4.70
	4h80_a	−11.30
	4h80_b	−11.40
	4l1o	−8.20
	4l2o_a	−8.50
	4l2o_b	−9.40
	VS1 [34]	−3.55
ALDH7A1	4zuk_a	−11.60
	4zuk_b	−12,20
	4zul_a	−8.50
	4zul_b	−8.80
	4zvw_a	−10.00
	4zvw_b	−11.20
	4zvx	−9.00
	4zvy	−9.20
	VS1 [34]	−2.45
ABCB1	6c0v	−13.30
	6qex_a	−9.90
	6qex_b	−10.90
	7o9w_a	−10.60
	7o9w_b	−10.70
	9cr8	−9.60
	9ctf	−7.50
	Palbociclib [35]	−9.61
	GSK-1070916 [36]	−8,00
ABCC1	2cbz	−6.00
	4c3z	−7.40
	8vt4	−10.40
	Sulfinpyrazone [37]	−6.80
ABCC2	8jx7	−10.10
	8jxq	−11.10
	8jxu	−12.10
	8jy4	−10.40
	8jy5	−10.60
	MK-571 [38]	−7.10
	Probenecid [38]	−6.10
	Irinotecan [38]	−9.70
ABCC3	8hvh	−12.30
	8hw2	−11.00
	8hw4	−10.10
	8izp	−10.00
ABCC4	8bjf	−11.30
	8i4c	−11.00
	8xok	−11.80
	8xol	−11.10
	8xom	−11.10
	MK57 [39]	−11,15
ABCG2	6feq	−9.90
	6hbu	−11.30
	6vxh	−9.20
	6vxi	−9.30
	6vxj	−9.40
	Mitoxantrone [40]	−6.30
	Hoechst-33342 [40]	−8.90

The impact of bis(aminothiocarbonyl)disulfide (**4)** on cell viability was analyzed using WST-1 assay (#8038, ScienCell Research Lab., Carlsbad, CA, USA). The control (non-cancerous) bronchus epithelial cells HBEC3-KT and the NSCLC cells: A549, NCI-H1581, and their cisplatin-resistant variants were cultured in their respective media. Next, the mixture was transferred to a 96-well plate, and after 24  h, the medium was changed to a full medium containing compound **4** (3 µM, 5 µM, 10 µM, and 15 µM, data not shown). After 48 h of incubation, cell viability was tested using the WST-1 assay according to the manufacturer's protocol. Similar to our previous work, a concentration of 3 µM did not have a significant impact on either control HBEC3-KT (**4** IC50 48 h = 8.1  µM) (Figure 6D) or NSCLC cell viability (**4** IC50 48 h: A549 = 8.4  µM; A549CisR = 5.1 µM; NCI-H1581 = 6.1 µM; NCI-H1581CisR = 7.8 µM), was chosen for further evaluation regarding the potential sensitization of cisplatin-resistant cells.^18^ After 24 h of incubation with bis-(di-4-phenyl-benzylaminethiocarbonyl)disulfide (**4**) presented no statistically significant impact on NCI-H1581 and NCI-H1581CisR response to cisplatin. Whereas, a significant decrease in cisplatin tolerance was noticed in the case of A549 and A549 CisR (cisplatin IC50 = 150  µM, cisplatin + **4** IC50 = 80 µM) (Figure 6E and Supplementary data S2).

**Figure 7.**
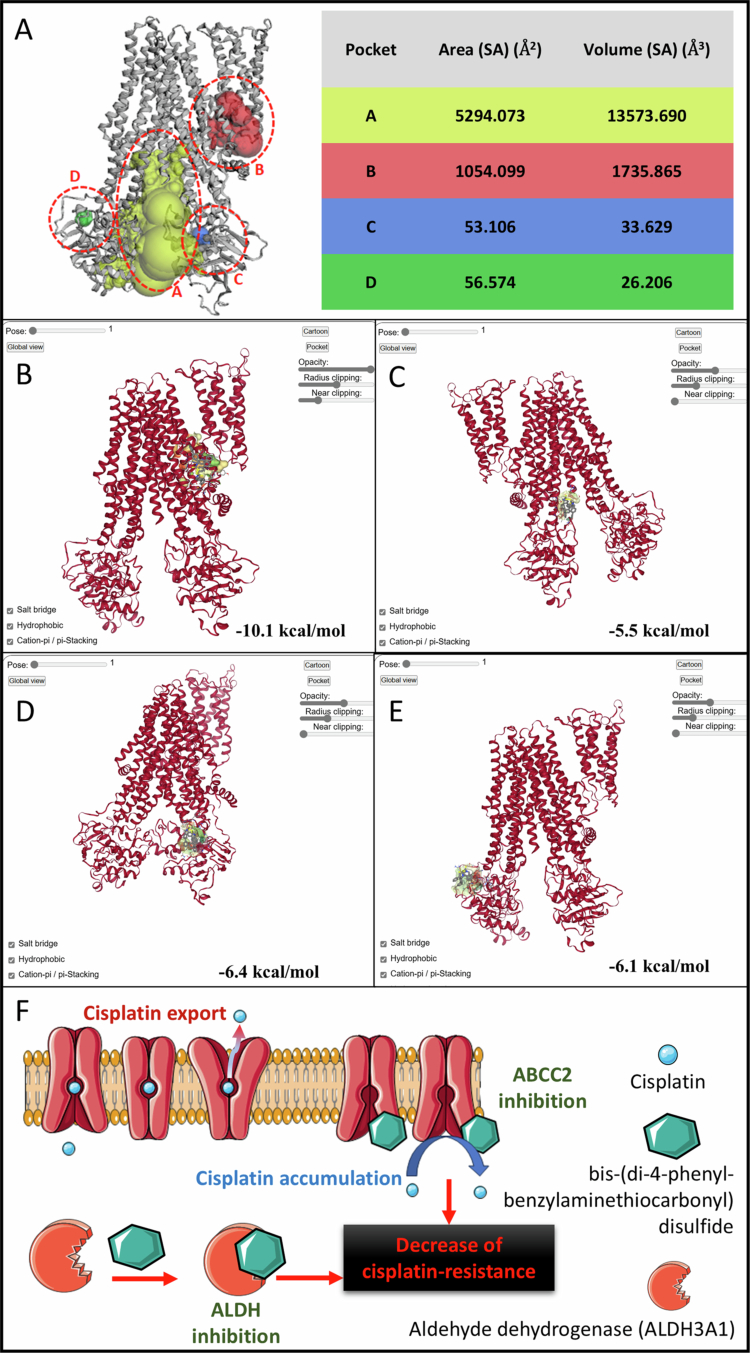
Bis-(di-4-phenyl-benzylaminethiocarbonyl)disulfide (4) affinity to ABCC2. Identification and physical characteristics of ABCC2 protein functional pockets were performed using the 8jx7 PDB structure and CASTpFold platform (A). The SeamDock platform and an 8jx7 PDB structure were used to test and visualize the affinity sites and calculate the respective affinity values. The highest affinity = −10.1  kcal/mol was observed for the transmembrane domain (TMD) (B). The inner part of the channel is formed by two TMDs with affinity = −5.5 kcal/mol (C). NBD1 and NBD2 with values of −6.4 and −6.1  kcal/mol, respectively (D and E). Schematic model of bis-(di-4-phenyl-benzylaminethiocarbonyl)disulfide interaction with ALDH active site and ABCC2, resulting in cisplatin accumulation, thus leading to cisplatin sensitization of cisplatin-resistant cells (F). Scheme created using images provided by Servier Medical Art (https://smart.servier.com/), licensed under CC BY 4.0.

To further explain this phenomenon, we analyzed the predicted ligand binding sites of product **4** to ABCC2 using an 8jx7 PDB structure (Figure 7). First, 315 ABCC2 functional protein pockets were identified and characterized using Computed Atlas of Surface Topography of the universe of protein Folds—CASTpFold (https://cfold.bme.uic.edu/castpfold/), with the most interesting presented in Figure 7A. ^41^ In contrast to ALDH3A1, ABCC2 is a transmembrane protein. Domains that are anchored in the cell membrane, even though they present affinity in mathematical models, may not be accessible in the cell for non-lipophilic compounds. The highest affinity site (−10.1 kcal/mol) regards the transmembrane domain (TMD), which is in close proximity to the cell membrane (Figure 7B). Therefore, in vivo, it can be inaccessible, even though the tested compound exhibits (to some extent) lipophilic properties. However, three additional important docking sites include the inner part of two TMDs, which create a channel for transported substrates (Figure 7C), and two nucleotide-binding domains (NBDs) (Figure 7D,E), which act as “motors” via ATP hydrolysis, providing conformational changes leading to substrate export.^42^ Interactions with those domains may decrease or completely inhibit their functions, leading to cisplatin cytoplasmatic level accumulation, which, combined with ALDH inhibition (via ALDH3A1), may lead to the sensitization of cisplatin-resistant cells to cisplatin (Figure 7F).

## Discussion

Lung cancer, especially NSCLC, remains one of the top causes of cancer-related deaths. Even though the treatment of advanced NSCLC may be evolving, the cisplatin-based treatment remains the foundation of treatment for most patients. Thus, in this respect, even discreet changes in the cisplatin administration procedure may contribute to an incremental improvement in patient survival.^43^ Although initially successful, treatments with chemotherapy, including cisplatin, often fail as a consequence of the acquisition of a chemoresistance phenotype by the primary tumor and/or the recurrence of chemo-resistant metastases. Since these instances develop after the first-line (L1) of treatment, chemoresistance is generally believed to appear in response to chemotherapy. However, alternative mechanisms of intrinsic chemoresistance in the chemotherapy-naïve setting may exist but remain poorly understood.^12^ Thus, current interest has shifted from analyzing single markers towards a holistic approach that takes into consideration the overall molecular landscape regarding the regulation of many, often complementary mechanisms, including physiological processes, that may help to predict patients' response to given treatment and prognosis for metastatic recurrence.^18^ Thus, a personalized approach for molecular.

Genetic variations in the efflux transporters involved in the pharmacokinetics of chemotherapeutics such as cisplatin or carboplatin, may alter their efficacy (leading to significant chemoresistance) and increase the risk of ADRs associated with chemotherapy.^23^ ABCC2 belongs to the multidrug resistance protein (MRP) family, consisting of 9 ABCC subfamily members (ABCC1-6, ABCC10-12), and was proven to be related to resistance to the platinum-based chemotherapeutics in NSCLC.^22^ ^,^ ^44^ Recently, pharmacovigilance analysis proved that patients carrying the TT genotype of ABCC2 rs717620 had a 14-fold higher risk of developing hepatic ADRs associated with elevated alkaline phosphatase (ALP) levels.^23^ Molecular mechanisms underlying NSCLC heterogeneity hold significant potential for clinical translation. Identifying malignant subpopulations and characterizing their distinct immune and metabolic features may provide novel insights for the precise diagnosis and prognostic assessment of lung cancer.^45^ Furthermore, MRP proteins, as potent drug exporters, have been extensively studied for several decades; their mRNA expression level is often upregulated as an adaptive response upon exposure to cytotoxic agents, as well as during epithelial-to-mesenchymal transition (EMT) via direct interaction of EMT-triggering transcription factors with E-box sequences in the ABC protein promoter region.^1^ ^,^ ^42^ ^,^ ^46^ Interestingly, ALDH3A1, which, in our analysis, is significantly upregulated in a specific sub-population of NSCLC to the level comparable with adjacent tissue, was proven to be related to the EMT and thus might be essential for the cells to maintain stemness.^47^

Our previous studies showed that isothiocyanates are strong anti-cisplatin-resistant agents. ITCs are low-molecular-weight, natural, organic composites characterized by a pungent odor, with the general formula R–NCS. They are found in cruciferous vegetables, such as radish, horseradish, wasabi, broccoli, or brussels sprouts, and are produced by the reaction of glucosinolates with myrosinases.^48^ Thus, to validate whether ALDH3A1 may not only be one of the most important predictors for NSCLC therapy outcome but also a target for anti-cisplatin-resistant therapy, we have synthesized bis-(di-4-phenyl-benzylaminethiocarbonyl)disulfide (**4**). This new compound **4** was obtained by combining two previously tested 1-(isothiocyanatomethyl)-4-phenylbenzene, which presented the highest affinity for ALDH3A1 (as assessed using AutoDockTools), via an S‒S bond.^49^ Disulfide bonds are very stable with a dissociation energy of 251 kJ/mol (approximately 40% weaker than a typical C‒C or C‒H bond). Thiol deprotonation is highly unfavorable at physiological pH (with a typical p*K*_a_ of a solvent-exposed thiol group of 8.3), and S-S breaking can be mediated by various enzymes, such as thioredoxins in the thiol‒disulfide exchange reaction.^50^ Obtained bis-(di-4-phenyl-benzylaminethiocarbonyl)disulfide (**4**) decreased cisplatin resistance of A54CisR, but not NCI-H1581. This fact is most likely due to the different mechanisms being predominantly involved in cisplatin resistance across the tested cell lines. In the case of AC NSCLC, the protein level of ALDH3A1 is significantly higher in A549CisR compared to the parental, non-cisplatin-resistant variant, whereas in NCI-H1581 and NCI-H1581CisR, its level is below the detection rate. Additionally, our findings prove that ALDH7A1, which is also a potent target for the tested ITCs, is less important for cisplatin resistance in the tested LCLC cell line. Despite its upregulation, in the case of NCI-H1581CisR, we did not observe any decrease in cisplatin resistance upon bis-(di-4-phenyl-benzylaminethiocarbonyl)disulfide (**4**) treatment. A549CisR cells exhibit increased sensitivity to electrophilic compounds such as bis-(di-4-phenyl-benzylaminethiocarbonyl)disulfide compared to their cisplatin-sensitive counterparts (A549), as well as both NCI-H1581 variants. This selective cytotoxicity is most probably linked to resistance-associated metabolic reprogramming, including the upregulation of aldehyde dehydrogenase isoforms such as ALDH3A1, which contributes to the detoxification of reactive aldehydes, redox balance, and tumor progression in NSCLC. Cisplatin-resistant cells, therefore, become highly dependent on ALDH3A1 and glutathione-mediated antioxidant systems to survive elevated basal oxidative stress. This adaptation creates a state of heightened metabolic and redox vulnerability. Bis-(di-4-phenyl-benzylaminethiocarbonyl)disulfide most probably disrupts this adaptive state by depleting glutathione and impairing detoxification capacity, leading to excessive accumulation of reactive oxygen species and lipid peroxides. Importantly, similar observations were recently presented in NSCLC models for sulforaphane (SFN) and phenethyl isothiocyanate (PEITC), which preferentially reduce viability and restore chemosensitivity in cisplatin-resistant cells through the induction of oxidative stress and ferroptotic or apoptotic cell death pathways.^51^ ^,^ ^52^ In contrast, cells that exhibit lower reliance on ALDH3A1-driven detoxification display reduced susceptibility to isothiocyanate-induced cytotoxicity.

The most important difference in the mediation of the cisplatin resistance mechanism concerns ABCC2—one of the most important cisplatin transporters.^42^ Bis-(di-4-phenyl-benzylaminethiocarbonyl)disulfide (**4**) presents an extremely high affinity to the ABCC2 and thus may increase the intracellular concentration of cisplatin. Importantly, the predicted docking sites consist of highly conserved (for all ABC proteins) nucleotide-binding domains—NBD1 and NBD2, which mediate ATP hydrolysis that drives the transport cycle, and the inner part of the formed transporting channel between transmembrane domains.^42^ This fact suggests that this inhibitory effect may also be observed in other (not tested) ABC proteins, which export vast amounts of different anticancer drugs, such as cisplatin, irinotecan, vinblastine, vincristine, doxorubicin, or methotrexate (to name a few), used in the therapy against many cancer types.^1^ ^,^ ^42^

The greatest limitation of this study is data availability and correctness, or, rather, consistency of data description. We based our data analysis on already existing datasets of patient transcriptomic data and our collection of NSCLC cell lines. However, the majority of GEO data regarding NSCLC consists of AC and SCC subtypes, omitting LCLC. On the other hand, our collection of NSCLC cell lines consists of AC and LCLC subtypes (and their respective cisplatin-resistant variants). Furthermore, *in vitro*-obtained models of chemoresistance may not fully reflect patient mechanisms, as they omit drug metabolism, bioavailability, and transport across patients' organs.^1^ ^,^ ^21^ Additionally, the description of the NSCLC stages in the case of some patients may not be fully correct, as, despite the experienced team effort, it is prone to human error. Binary classification of clinical samples cannot ignore that disease progression is likely more continuous than discrete, and from both clinical and molecular perspectives, no clear borders between disease stages may be observed.^53^ Furthermore, despite repeated optimization efforts, several Western blot images remained visually suboptimal owing to the technical limitations of the method. Nevertheless, semi-quantitative interpretation was based on digital densitometric analysis of pixel intensity rather than visual inspection alone. Importantly, the key data concerning ABCC2 and ALDH isoforms were supported by reproducible results and complementary experimental approaches, minimizing the risk of overinterpretation.

## Conclusion

Owing to phenotypical and molecular differences, lung cancer is highly heterogeneous, and various NSCLC subtypes respond differently to given therapy schemes. We have found significant subpopulations of NSCLC patients characterized by high expression level of ABCC2, which correlates with ALDH3A1, most probably leading to the intrinsic/acquired cisplatin-resistance, suggesting that molecular categorization is essential. Additionally, bis-(di-4-phenyl-benzylaminethiocarbonyl)disulfide (**4**) decreases cisplatin resistance in ALDH3A1- and ABCC2-overexpressing A549 cisplatin-resistant cells but not in a cisplatin-resistant variant of NCI-H158, which present low level of these two proteins. Additionally, we believe that in future clinical practice, this could be analyzed using the tumor/non-tumor ABCC2 and ALDH3A1 mRNA expression ratios. Further studies are needed to test any possible beneficial impact of bis-(di-4-phenyl-benzylaminethiocarbonyl)disulfide on the cytotoxicity of other drugs and on other cancer types.

## Materials and methods

### Cell culturing and in vitro induction of drug resistance

Cell culture was performed as previously described.^18^ Briefly, the bronchus epithelial hTERT-immortalized cell line HBEC3-KT (CRL-4051) and the lung cancer cell lines A549 (CRM-CCL-185) and NCI-H1581 (CRL-5878) were obtained from the ATCC (Manassas, VA, USA). All the cell lines that underwent short tandem repeat (STR) analysis were provided by ATCC, proving their authenticity. The cisplatin-resistant variants of both cancer cell lines were established by constant culturing in the presence of increasing concentrations of cisplatin to a final concentration of 10 μM over approximately 6 months. HBEC3-KT were cultured in Airway Epithelial Cell Basal Medium #PCS-300-030 (ATCC, Manassas, VA, USA) supplemented with Bronchial Epithelial Cell Growth Kit #PCS-300-040 (ATCC, Manassas, VA, USA), penicillin (10 units/mL), and streptomycin (10 µg/mL) (Biowest, Nuaillé, France). A549 and A549CisR cells were cultured in Ham's F12-K medium (Corning, Manassas, VA, USA), NCI-H1581 and NCI-H1581CisR cells were cultured in DNEM/HAM F-12 (Corning, Manassas, VA, USA) in a 90–95% humidified atmosphere of 5% CO2; the media were supplemented with 10% heat-inactivated fetal bovine serum (FBS) (Biowest, Nuaillé, France) and the antibiotics streptomycin, penicillin (Biowest), and primocin (InvivoGen, San Diego, CA, USA). The cells were plated in 25 cm2 cell culture flasks and subcultured before reaching confluency using Accutase (Biowest). The cells were frequently tested for mycoplasma using the Mycolor One-Step Mycoplasma Detector Kit #D201-02 (Vazyme, Nanjing, China). Cell counting and cell viability (live/dead cell ratio) were performed using 0.4% Trypan blue and an automatic cell counter.

### Reagents and chemicals

All the compounds were >95% pure by HPLC. Cisplatin [cis-diammineplatinum(II) dichloride] was obtained from Sigma-Aldrich (St. Louis, MO, USA) #15663-27-1; WST-1 [2-(4-Iodophenyl)-3-(4-nitrophenyl)-5-(2,4-disulfophenyl)-2H-tetrazolium] was purchased from ScienCell (Research Lab., Carlsbad, CA, USA) freshly made, #8038; RIPA lysis buffer was purchased from VWR Chemicals, #N653-100 mL; protease inhibitor was purchased from Thermo Fisher Scientific (Waltham, MA, USA), #PIER87785; BCA was purchased from Thermo Fisher Scientific, #PIER23225. Primary antibodies:, ABCC1 #VMA00330 BioRad (Hercules, CA, USA), ABCC1, ABCC2 ABCC3 ABCC5, α-Tubulin #NB100-690H Novus Biologicals (Littleton, CO, USA), ALDH3A1 #PA5-80332 Thermo Fisher Scientific, and ALDH7A1 #MA5-29028 Thermo Fisher Scientific. The secondary HRP-conjugated antibodies were purchased from Santa Cruz Biotechnology.

### Synthesis of bis-(di-4-phenyl-benzylaminethiocarbonyl)disulfide (4)

General information: NMR spectra were measured on a Bruker Avance II Plus 700 MHz (Bruker Corporation, Billerica, MA, USA) spectrometer in a CDCl_3_ solution. Chemical shifts (*δ*) are reported in ppm, and coupling constants (*J*) are reported in Hz. ^1^H and ^13^C NMR spectra were referenced according to the residual peak of the solvent based on literature data. ^13^C NMR spectra were proton-decoupled. LC‒MS analysis was performed using a UHPLC VanquishFlex liquid chromatography from Thermo Fisher Scientific® and an ESI-Q-TOF Impact II mass spectrometer from Bruker®. The sample for analysis was diluted in a mixture of water:acetonitrile (1:1) supplemented with 0.1% formic acid. All reagents used for analysis were of LC‒MS purity. The monoisotopic mass of the products obtained was confirmed by high-performance liquid chromatography‒mass spectrometry coupled analysis. The chromatographic separation of the compounds was carried out in a reversed-phase system using a Bruker® Intensity Solo 2 column, dimensions: 100 × 2.1 mm, filling C18, pore size 100 Å, column temperature 30 °C, injection size 2  μl, mobile phase flow rate 0.3 ml/min, and gradient elution according to the following ratio: water (component A)/acetonitrile (component B), (both components with the addition of 0.1% formic acid): (A/B) 0–2 mm 70/30, 2–10 min 50/50, 10–15 min 0/100, 15–20 min 0/100, 20–25 min 70/30, and 25–30 min 70/30. Flash chromatography was performed using a glass column packed with Baker silica gel (30–60 m). For TLC, silica gel with a 254 nm indicator on Al foils (Sigma-Aldrich, St. Louis, MO, USA) was used. All reagents (4-phenylbenzylamine and biphenyl-4-carboxaldehyde) and solvents were purchased from Merck Life Science (Poznań, Poland) and used as obtained.

Synthesis of di-(4-phenylbenzyl)amine 3.

**Figure:**
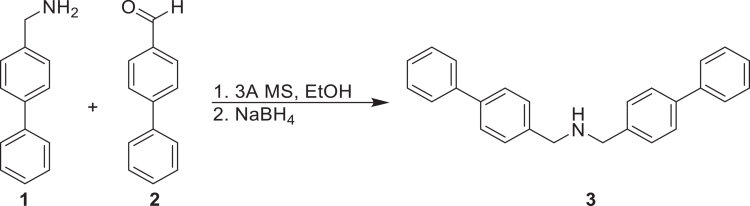


In the flame-dried 50 mL round-bottom flask, 4-phenylbenzylamine (**1**, 0.550 g, 3 mM, 1 eq) and biphenyl-4-carboxaldehyde (**2**, 0.546 g, 3 mM, 1 eq) were dissolved in absolute ethanol (10 mL), and 3 Å MS was added (0.6 g). After 3 min of mixing at room temperature, a white precipitate was formed, and CH_2_Cl_2_ (6 mL) was added to dissolve it. The reaction was stirred at room temperature for 2 h and then controlled by NMR. ^1^H NMR showed >95% conversion of the aldehyde into the imine. Next, NaBH_4_ (0.170 g, 4.5 mM, 1.5 eq) was added in two portions, and the stirring was continued at room temperature. After 2 h, the precipitate and MS were filtered, and the filtrate was evaporated under reduced pressure. The crude product **3** was purified by recrystallization from hot EtOAc/hexane and obtained as a white solid of **3** (0.446 g, 1.3  mM, 43%). The analytical data of **3** are in agreement with those reported previously in the literature.^32^

^**1**^ **H NMR** (700 MHz, CDCl_3_): *δ* 7.61–7.58 (m, 8 H, 8 × C*H*_Ar_), 7.46–7.43 (m, 8 H, 8 × C*H*_Ar_), 7.36–7.33 (m, 2 H, 2 × C*H*_Ar_), 3.90 (s, 4 H, 2 × C*H*_2_), 1.65 (bs, 1 H, N*H*). ^**13**^ **C NMR** (176 MHz, CDCl_3_): *δ* 141.1, 140.0, 139.5, 128.8, 128.7, 127.3, 127.2, 53.0.

Synthesis of bis-(di-4-phenyl-benzylaminethiocarbonyl)disulfide (4).

**Figure:**
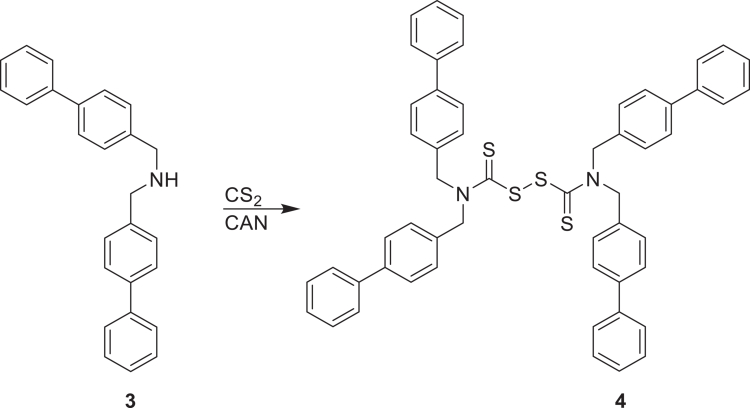


Compound **3** (0.350 g, 1 mM, 1eq) was dissolved in THF (2 mL) in a 25 mL round-bottom flask, and then CS_2_ (0.1 mL, 1.2 mM, 1.2 eq) and a catalytic amount of ceric ammonium nitrate (CAN, 0.3 mg) were added. The mixture was stirred for 10 min with a stopper on the flask and 8  h without it at room temperature. After this time, THF was removed under reduced pressure to obtain the crude product **4**. Next, CH_2_Cl_2_ (4 mL) and 1 N HCl (0.5 mL) were added, and the reaction was stirred for 10 min. The aqueous phase was separated and extracted by CH_2_Cl_2_ (2 × 4 mL). The combined organic layers were dried under anhydrous MgSO_4_. The solvent was evaporated under reduced pressure, and the final compound **4** was isolated using flash chromatography (hexane: EtOAc 3:1) with a 12% (0.1 g, 0.12  mM) yield.

Purity 99%, *t*_R_ = 19.0 min. ^**1**^ **H NMR** (700 MHz, CDCl_3_): *δ* 7.67–7.66 (m, 4 H, 4 × CH_Ar_); 7.61–7.59 (m, 12 H, 12 × CH_Ar_), 7.54–7.53 (m, 4 H, 4 × CH_Ar_), 7.46–7.44 (m, 12 H, 12 × CH_Ar_), 7.38–7.36 (m, 4 H, 4 × CH_Ar_), 5.47 (s, 4 H 2 × CH_2_), 5.31 (s, 4 H 2 × CH_2_). ^**13**^ **C NMR** (176 MHz, CDCl_3_): *δ* 196.4, 141.4, 141.0, 140.7, 140.5, 134.0, 133.3, 128.9, 128.5, 128.1, 127.9, 127.7, 127.6, 127.5, 127.2, 59.0, and 55.0. **HRMS**: *m/z* [M + H]^+^ calcd for C_54_H_45_N_2_S_4_^+^ 849.2460, found 849.2268. New compound.

^1^H and ^13^C NMR spectra of compounds **3** and ^1^H and ^13^C NMR spectra, chromatogram UV–Vis and HR-MS spectrum of **4** are presented in Supplementary data S3–S8.

### Molecular modeling

Identification and dimension characteristic of the ABCC2 functional pocket was performed using the Computed Atlas of Surface Topography of the universe of protein Folds - CASTpFold platform (https://cfold.bme.uic.edu/castpfold/).^41^ Affinity calculations were performed analogously to our previous paper using software dedicated to molecular docking: AutoDockTools v.1.5.6 (La Jolla, California, USA), included in the MGLTools 1.5.6 (La Jolla, California, USA) package, and AutoDock Vina 1.1.2 (La Jolla, California, USA) and a PC equipped with a 12-core/24-thread AMD Ryzen 9 3900X processor.^49^. Briefly, files describing proteins were downloaded in pdb format from the RCSB Protein Data Bank (https://www.rcsb.org/) and then converted using AutoDockTools to the pdbqt format required by AutoDock Vina.

The compound structures were drawn in ACD/ChemSketch (Toronto, Ontario, Canada) (Freeware), and then 3D structure optimization was performed. The structures were saved in .mol (MDL MOL) format. Next, the structures in .mol format were converted to .pdb (Protein Data Bank) format using OpenBabelGUI. The .pdb format files were used to model docking in the Vina package.

Visualization of docking sites was performed using SeamDock Open software (https://bioserv.rpbs.univ-paris-diderot.fr/services/SeamDock/#usage).

### Cell viability assay

Cisplatin IC50 values for NCI-H1581 and NCI-H1581CisR were determined to be analogous to our previous work on A549/A549CisR using WST-1 assay.^18^ In brief, the cells (2 × 10^5^ cells/mL) were seeded on 96-well culture plates and left for 24 h. Next, parental and resistant tumor cells were incubated in 100 µL of fresh medium containing different drug concentrations. Next, after 48 h of incubation, 10 µL of WST-1 reagent (ScienCell, Research Lab., Carlsbad, CA, USA), freshly made, #8038, was added for 2 h. The calculation of cell viability was done by OD450–OD630 nm using the BioTek ELx800 multimode microplate reader.

### Microscopic visualization

The cells were grown on 6-well plates (Nunc, Thermo Fisher Scientific, Waltham, MA, USA). Next, cisplatin and/or bis-(di-4-phenyl-benzylaminethiocarbonyl)disulfide were added for 24 h. Adherent cells (A549 and A549CisR) were washed 3× with PBS to remove dead cells, in the case of NCI-H1581 and NCI-H1581CisR, which are mixed: adherent and suspension, suspension cells were gently removed without washing. Finally, fresh medium was added, and images were acquired using an Olympus IX53 microscope (Tokyo, Japan) and cellSens Microscope Imaging Software (https://evidentscientific.com/).

### Calculation and visualization based on RadViz algorithm

Calculation and visualization of the RadViz algorithm and the linear projection component of the RadViz algorithm were delivered via the Orange Data Mining 3.31.1 platform (https://orangedatamining.com/). This algorithm is based on the systematic method for evaluating low-dimensional projections of high-dimensional data with respect to their ability to visually separate classes introduced by Gregor Leban and named the VizRank algorithm, in which the goal is to find a projection from $R^{d}\;$ for a given dataset $X\in R^{n\times d}$ with class labels y in a 2D space that maximizes class separability when visualized.^24^ This complicated mechanism could be explained using a simple physical analogy with multiple springs attached with one end to the attribute (in this instance, the chosen gene) and with the other end to the data point inside the visualization space. The stiffness of each spring in terms of Hooke's law is determined by the corresponding attribute value (in this case, the chosen gene expression level); thus, the greater the attribute value, the greater the stiffness, and the data point is drawn closer to “stronger” attributes. In the aftermath, the data point is placed where the sum of all spring forces equals 0. This simple, yet not over-simplified, approach is distribution-independent and can be applied to data obtained using different methods, requiring almost no data preparation.^24^ ^,^ ^31^ A linear projection in VizRank/RadViz is defined as Z=XW , where X is the original data matrix, $W\in R^{d\times k}$ is a projection matrix (usually k=2 ), and $Z\in R^{n\times k}$ is the projected data. Each column of W represents a linear combination of original features. In practice, the linear projection component, instead of placing multiple attributes in the circumference of the graph, uses the Cartesian coordinate system, placing the best-matching data representing the highest projection fit in the I quadrant, where (in typical data depiction) both X and Y axes present values > 0.^24^ ^,^ ^31^

### Western immunoblotting

Total protein was extracted from cells using ice-cold M-PER Mammalian Protein Extraction Reagent #78501 supplemented with the Halt protease inhibitor cocktail (Thermo Scientific, Waltham, MA, USA), and the soluble protein fraction was collected through centrifugation. The protein concentrations in the cell lysates were measured via the BCA method (Pierce/Thermo Scientific, Waltham, MA, USA) and were equalized between samples. Protein (40 µg) from whole-cell lysates was fractionated on SDS–PAGE gels and transferred to a PVDF or nitrocellulose membrane (Bio-Rad, Hercules, CA, USA). Transfer efficiency and loading were confirmed by reversible staining of the membrane with Ponceau S solution (Sigma-Aldrich, UK) following protein transfer. The membranes were blocked at room temperature with a blocking buffer (BioRad, Hercules, CA, USA). Primary antibodies were added in 1:1000–1:5000 dilution and incubated for 1 h at RT. The membranes were washed 3 × 15′ with TBST and incubated with a secondary horseradish peroxidase (HRP)—labeled antibody for 1 h at RT (1:2000). The membranes were washed in 3 × 15′ with TBST following incubation with secondary antibodies. Bound antibody complexes were detected and visualized using Clarity Western ECL Substrate (Bio-Rad, Hercules, CA, USA). Representative blot images from three independent experiments (*n* = 3) are shown and were subsequently subjected to densitometric analysis using ImageJ 1.53k (Java 1.8.0_172) software. Densitometric normalization was performed in two steps. First, the intensity of each band was normalized to that of the corresponding protein loading control. Subsequently, for visualization purposes, the signal intensity of each protein band in A549 cells was defined as 100%, and the remaining values were calculated and are presented as the percentage change relative to A549 (Supplementary data S1). Furthermore, to facilitate interpretation of the observed differences in protein expression between the respective parental and resistant subvariants, the percentage fold change was calculated and included in the Results section.

### Microarray data processing and analysis

Gene Expression Omnibus (GEO) database (http://www.ncbi.nlm.nih.gov/geo/ (accessed on 10 July 2024)) gene expression profiles were downloaded with accession numbers: GSE102287 and GSE43580 and analyzed with GEO2R online tool which uses the online analytical tool, which uses the R language (# Version info: R 4.2.2, Biobase 2.58.0, GEOquery 2.66.0, limma 3.54.0) (https://www.ncbi.nlm.nih.gov/geo/geo2r/ (accessed on 10 July 2022)). The calculation and visualization of gene expression were performed using Jasp 0.16.0.0 software (https://jasp-stats.org/ (accessed on 10 July 2024)), similar to our previous work.^54^ ^,^ ^55^ The mRNA expression levels of the chosen genes in the A549 and NCI-H1581 cell lines were verified using the GENEVESTIGATOR platform (https://genevestigator.com/) (accessed on 10 April 2025). Outlier data were not removed to identify subgroups. GSE102287 data are already log2 transformed, and GSE43580 were log2 transformed to calculated Z score using the Z = (x−μ)/σ (where: x = expression value of the gene in one sample, *μ* = mean expression of that gene across samples and *σ* = standard deviation across samples) in R. Probe-to-gene mapping was conducted by the original authors of each dataset and further verified using hgu133plus2.db.

### Proteome analysis of NSCLC cancer samples

The analysis of chosen ALDH and ABC protein levels in NSCLC cancer samples (AC and SCC) was performed using “The Human Protein Atlas” platform, analogous to our previous work.^25^ All the data in the HPA resource are open-access (https://www.proteinatlas.org).^26^ ^,^ ^27^ Antibody used for histopathology staining: anti-ALDH3A1 - HPA051150, anti-ALDH7A1 - HPA053675, anti-ABCC2 -HPA071145.

### Survival probability analysis

The survival rate for patients presenting the high and low expression of genes coding ALDH3A1, ALDH7A1, and ABCC2 proteins was analyzed in NSCLC patients using TCGA data and the TIMER2.0 platform (http://timer.cistrome.org/, accessed on 10 August 2023).^28^

### Statistics

Every *in vitro* experiment was conducted in a minimum of *n* = 3 repeats. The *n* value of each microarray is stated in the results section. Statistical evaluation was performed using a paired *t*-test for paired data, the normality test (Shapiro–Wilk), followed by Student's *t*-test (in the case of normally distributed data), or one-way ANOVA post hoc test (Tukey), and the results were visualized using Jasp 0.16.0.0 software. Calculations and graphs were performed using Orange data mining 3.31.1 software and JASP 0.16.0.0 software; *p* values < 0.05 were considered statistically significant for all analyses: *p < 0.05; **p < 0.005; ***p < 0.001, NS-not statistically significant. Pearson's linear correlation analysis was performed using JASP 0.16.0.0 software, with the Pearson correlation coefficient presented as color intensity and numerical values on the correlation matrix. The correlation statistical values are as follows: *p < 0.05; **p < 0.005; ***p < 0.001, no indication—not statistically significant. The Benjamini and Hochberg adjusted *p*-value (Padj.) from the stats package in R, which is the transformation of the *p*-value using the false discovery rate (FDR)—[P_values, method = “fdr”], was used to test statistically significant differently expressed genes (DEGs).^56^

### Schematic images

Schematic images were created using graphics provided by Servier Medical Art (https://smart.servier.com/), licensed under CC BY 4.0.

## Supplementary Material

Supplementary Material Supplementary .docx

## Data Availability

The data used to support the findings of this study are available at the GEO database http://www.ncbi.nlm.nih.gov/geo/ GSE102287: https://www.ncbi.nlm.nih.gov/geo/query/acc.cgi?acc=GSE102286 and GSE43580: https://www.ncbi.nlm.nih.gov/geo/query/acc.cgi?acc=GSE43580. Images of ALDH3A1, ALDH7A1, and ABCC2 proteins in NSCLC tissue samples are available at https://v20.proteinatlas.org/ENSG00000108602-ALDH3A1/pathology/lung+cancer#img, https://v20.proteinatlas.org/ENSG00000164904-ALDH7A1/pathology/lung+cancer#img, and https://v20.proteinatlas.org/ENSG00000023839-ABCC2/pathology/lung+cancer#img. Additional data are available from the corresponding author upon request.
