# Supplementary material for: Bis-(di-4-phenyl-benzylaminethiocarbonyl)disulfide sensitizes ABCC2/ALDH3A1 overexpressing NSCLC cells to cisplatin
Source: Cancer Biol Ther. 2026 Jul 20;27(1):2683169. doi: 10.1080/15384047.2026.2683169 (PMC13387110; doi:10.1080/15384047.2026.2683169)
Supplement: Supplementary Material — Supplementary .docx [file KCBT_A_2683169_SM5450.docx]

**Bis-(di-4-phenyl-benzylaminethiocarbonyl)disulfide Sensitizes ABCC2/ALDH3A1 overexpressing NSCLC cells to Cisplatin**

**Jolanta Kryczka^1^, Jakub Kryczka^2^*, Łukasz Janczewski^3^, Sho Shimida^4^,** [**Andrzej Frączyk**](https://pubmed.ncbi.nlm.nih.gov/?sort=date&term=Fr%C4%85czyk+A&cauthor_id=35955773)**^5^, Beata Kolesińska^3^, Joanna Boncela^2^, Ewa Brzeziańska-Lasota^1^**

^1^ Department of Biomedicine and Genetics, Medical University of Lodz, 92-213 Lodz, Poland.

^2^ Laboratory of Cell Signalling, Institute of Medical Biology, Polish Academy of Sciences, 93-232 Lodz, Poland.

^3^ Institute of Organic Chemistry, Faculty of Chemistry, Lodz University of Technology, 90-924 Lodz, Poland.

^4^ School of Integrated Design Engineering, Graduate School of Science and Technology, Keio University, 3-14-1 Hiyoshi, Kohoku, Yokohama 223-8522, Japan

^5^ Institute of Applied Computer Science, Lodz University of Technology, 90-537 Lodz, Poland.

**Supporting Information**


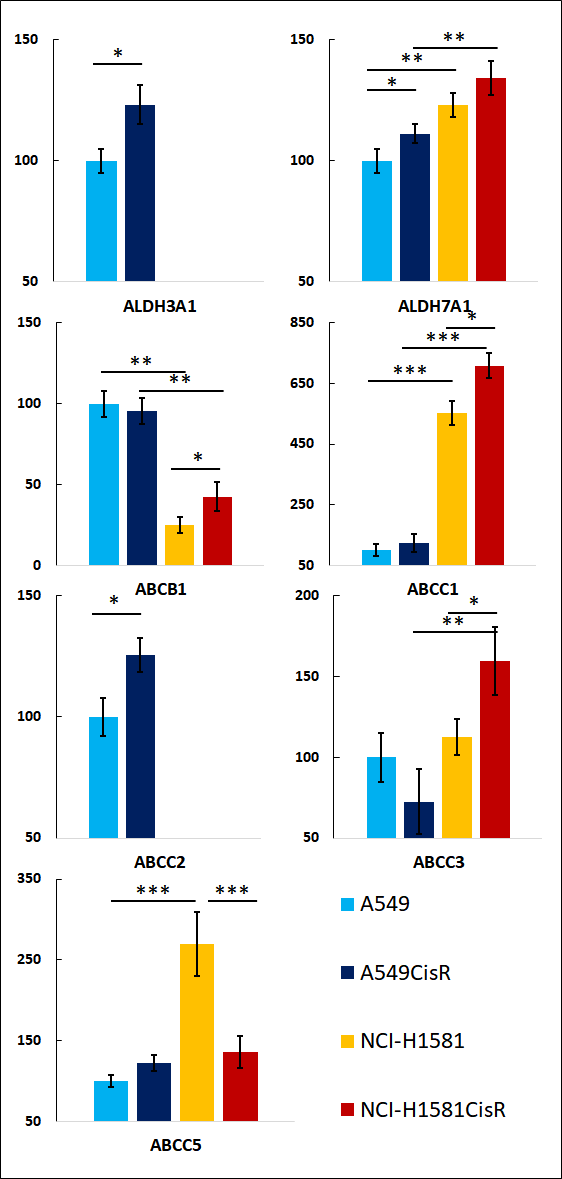


Fig S1. Densitometric analysis of representative blot images from three independent experiments (n = 3) was performed using ImageJ 1.53k (Java 1.8.0_172). Band intensities were normalized to the corresponding loading controls and expressed relative to A549 cells, which were set to 100% (Y axis) for visualization purposes. Statistical analysis was performed using the t-test, comparing respective parental vs resistant variants, as well as A549 vs NCI-H1581 and A549CisR vs NCI-H1581CisR cells * p < 0.05, ** p < 0.005, and *** p < 0.001


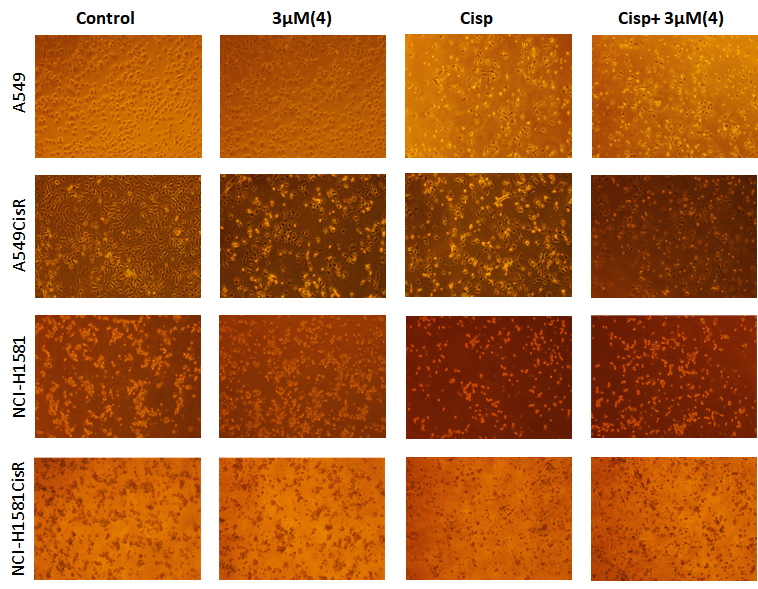


Fig S2. Visualization of 3µM bis-(di-4-phenyl-benzylaminethiocarbonyl)disulfide (**4**) and cisplatin impact on A549, A549CisR, NCI-H1581 and NCI-H1581CisR viability.

^1^H and ^13^C NMR spectra of compound **3** and ^1^H and ^13^C NMR spectra, chromatogram UV-Vis and HR-MS spectrum of compound **4**

Fig S3. ^1^H NMR of compound **3** (700 MHz, CDCl_3_).

Fig S4. ^1^H NMR of compound **3** (176 MHz, CDCl_3_).

Fig S5. ^1^H NMR of compound **4** (700 MHz, CDCl_3_).

Fig S6. ^13^C NMR of compound **4** (176 MHz, CDCl_3_).

Fig S7. Chromatogram UV-Vis of compound **4**.

Fig S8. HR-MS spectrum of compound **4**.
